# Supplementary material for: Iron deficiency and common neurodevelopmental disorders—A scoping review
Source: PLoS One. 2022 Sep 29;17(9):e0273819. doi: 10.1371/journal.pone.0273819 (PMC9522276; doi:10.1371/journal.pone.0273819)
Supplement: S1 File — Studies investigating the association of ADHD and ID. (DOCX) [file pone.0273819.s002.docx]

**S2 and S3 Tables. Studies investigating the association of ADHD and ID**

** indicates a statistically significant association; ---- indicates no information provided. Abbreviations: ADHD-NOS: not otherwise specified, criteria for ADHD was not met, but exhibited ADHD-like behaviours; BI: brain iron; CBCL: Child Behaviour Checklist; CPRS: Conners’ Parent Rating Scale; CPRS-R: revised Conners’ Parent Rating Scale; CRS: Conners’ Rating Scale; CTRS: Conners’ Teacher Rating Scale; ECI-4: Early Childhood Inventory-4; FEP: free erythrocyte protoporphyrin; GDD: global developmental delay; Hb: hemoglobin; HBQ: MacArthur Health and Behaviour Questionnaire; Hct: hematocrit; IA: internationally adopted; ID: iron deficiency; IDA: iron deficiency anemia; K-SADS-PL: Kiddie-Schedule for Affective Disorders and Schizophrenia: Lifetime Version; K-SADS-E: Kiddie-Schedule for Affective Disorders and Schizophrenia: Epidemiological Version; MCV: mean corpuscular volume; MCHC: mean corpuscular hemoglobin concentration; MINI Kid: Mini-International Neuropsychiatric Interview for Children and Adolescents; Mo: months; RLS: restless legs syndrome; RDW: red cell distribution width; SCID-I: Structured Clinical Interview for DSM-IV Axis Disorders; SDQ: Strengths & Difficulties Questionnaire; SF: serum ferritin; SFI: sTfR/log ferritin index; SI: serum iron; SNAP-IV: Swanson, Nolan, and Pelham – IV questionnaire; SWTD: sleep wake transition disorder; T-DSM-IV-S: Turgay DSM-IV-Based Child and Adolescent Behaviour Disorders Screening and Rating Scale; TF: transferrin; TSAT: transferrin saturation; TIBC: total iron binding capacity; WURS: Wender Utah ADHD Rating Scale; YSR: Youth Self Report

| **S2 Table**  **ADHD & ID positive association**  **N=22** | | | | | | | | | | |
| --- | --- | --- | --- | --- | --- | --- | --- | --- | --- | --- |
| **Case control studies**  **N=12** | | | | | | | | | | |
| **Ref.** | **Country** | **Study population** | | | **ID markers** | | **ADHD Instrument** | **Results** | | |
|  |  | Cases  Controls  (n) | Male  Female  (n) | Age (y)  Range  Mean | Iron-  related  (cut-off values; N/A= no cutoff value specified) | RBC-related |  | ID & ADHD | ID & ADHD severity |  |
| Konofal et al  2004 [32] | France | 53  ADHD  27 controls | 45  8  20  7 | 4-14  ----  5-15  ---- | SF**  (<15-30 ug/L)  SI | Hb  Hct | CPRS | SF lower in ADHD (84%) vs controls (18%) (p<0.001) | Low SF associated with more severe ADHD symptoms (p<0.02) and greater cognitive deficits (p<0.01) |  |
| Konofal et al  2007 [36] | France | 10  ADHD  12 ADHD & RLS  10  Controls | 9  1  8  4  7  3 | 5-8  6.7  5-8  7.3  5-8  7.0 | SF**  (N/A)  SI | Hb  Hct | CPRS | SF significantly lower in ADHD (p<0.0005) |  |  |
| Juneja et al  2010 [31] | India | 25  ADHD  25 controls | 21  4  ----  ---- | 6-14  8.4  6-14  7.9 | SF**  (<12 ug/L) | Hb  Hct | CPRS  CTRS | SF significantly lower in ADHD (p<0.001) | Negative correlation between oppositional subscore of CRS and SF |  |
| Mahmoud et al  2011 [13] | Egypt | 58  ADHD  25 controls | 26  32  12  13 | 5-13  8.3  5-15  8.6 | SF**  (N/A) | Hb**  Hct | CPRS | SF (p=0.03) & Hb significantly lower in ADHD (p=0.04) along with zinc and magnesium levels |  |  |
| Cortese et al  2012 [39] | France | 18  ADHD  9  controls  9  psych. controls | 16  2  5  4  5  4 | 8-14  118.8 mo  8-14  120.8 mo  8-14  123.5 mo | BI**  SF**  (N/A)  SI | Hb | K-SADS-PL | SF significantly lower in ADHD (p<0.001); BI significantly lower in ADHD compared to healthy controls. No correlation between SF & BI |  |  |
| Bener et al  2014 [60] | Qatar | 630  ADHD  630  controls | 315  315  313  317 | 5-18  11.54  5-18  11.50 | SF**  (<15ug/L)  SI** | Hb** | CPRS  CTRS  SNAP-IV | SF, SI, & Hb significantly lower in ADHD (p<0.01) |  |  |
| Seleem et al  2014 [30] | Egypt | 30  ADHD  15 controls | 24  6  11  14 | 6-12  8.6  6-12  9.7 | SF**  (<30 ug/L)  SI  TIBC | Hb**  MCH  MCHC  MCV  RDW | CPRS  MINI Kid | SF (p=0.01) & Hb (p=0.009) significantly lower in ADHD | Negative correlation between SF and ADHD total & cognitive subscore of CPRS-R (p<0.05) |  |
| Percinel et al  2016 [35] | Turkey | 200  ADHD  100 controls | 127  72  60  40 | 7-15  10.95  5-18  11.0 | SF**  (N/A)  TIBC  SI | Hb  Hct  MCV  RDW | CPRS  CTRS  K-SADS-PL  T-DSM-IV-S | No significant difference in SF between children with ADHD & controls | Negative correlation between CPRS/CTRS hyperactivity subscale scores & SF (p<0.001) |  |
| Demirci et al  2017 [25] | Turkey | 83  ADHD  70 controls | 0  83  0  70 | 18-50  23.4  18-50  23.4 | SF**  (<15 ug/L)  TIBC**  SI** | Hb** | Adult ADD/ADHD Evaluation Scale  SCID-I  WURS | 18% IDA cases had ADHD vs 2.9% controls | IDA cases had higher WURS (p=0.002), SF & SI negatively, TIBC positively correlated with WURS |  |
| Abd El Naby et al  2018 [41] | Egypt | 25 ADHD  25 ADHD & epilepsy  25 epilepsy  25 controls | 19  6  17  8  13  12  13  12 | ----  4.0  ----  4.11  ----  4.25  ----  5.66 | SF**  (N/A) | ---- | ADD/  ADHD DSM IV based Diagnostic Screening and Rating Scale | SF significantly lower in ADHD compared to control, epilepsy, & ADHD & epilepsy groups. |  |  |
| Islam et al  2018 [40] | India | 119  ADHD  119  controls | 84  35  85  34 | 0-19  11.0  0-19  11.2 | SF**  (N/A)  SI**  TIBC** | Hb**  MCHC  **  MCV** | CPRS  CTRS | Iron & RBC markers significantly lower in ADHD. Children with IDA were at higher risk for ADHD (OR=3.82) |  |  |
| Sahu et al  2020 [38] | India | 35  ADHD  35 controls | 35  0  35  0 | 5-16  9.46  5-16  9.71 | sTfR  SF  (<16 ug/L)  SFI**  SI  TIBC  TSAT** | Hb | CPRS | Significantly lower levels of TSAT in the ADHD group (p=0.030). SFI >1.5 associated with ADHD (p=0.015) | Significant correlation between TSAT and CPRS (subscales: hyperactivity (p=0.045), global index (p=0.029), DSM IV-TR-hyperactive-impulsive (p=0.015)) |  |
| **Case series**  **N=8** | | | | | | | | | | |
| Oner et al  2008 *a* [33] | Turkey | 151  ADHD | 127  24 | 5-16  9.9 | SF**  (<12 ug/L) | Hb**  MCV**  RDW | CPRS  CTRS  K-SADS-PL |  | Negative correlation between CPRS/CTRS & SF. No significant correlation in cases with ADHD without comorbid conditions. |  |
| Oner et al  2008 *b* [29] | Turkey | 52  ADHD | 42  10 | 7-13  9.9 | SF**  (<12 ug/L) | Hb  MCV  RDW | CPRS  CTRS |  | Inverse correlation of CPRS and hyperactivity scores & SF |  |
| Cortese et al  2009 [14] | France | 68  ADHD | 56  12 | 6-14  9.1 | SF**  (<45 ug/L) | Hb | CPRS  K-SADS-PL |  | Inverse correlation between SWTD & CPRS score & SF |  |
| Calarge et al  2010 [27] | USA | 52  ADHD | 42  9 | 6-14  10 | SF**  (< 7ug/L &  < 30ug/L) | Hb  Hct  MCV**  RDW | SNAP-IV | 23% ID | Inverse correlation of total ADHD score and inattention, hyperactivity, & impulsive scores & SF. Inverse correlation stronger in previously medicated (e.g. psychotropic) children. |  |
| Oner et al  2010 [34] | Turkey | 118  ADHD | 97  21 | 7-14  9.8 | SF**  (N/A) | Hb  MCV  RDW** | CPRS  CTRS  K-SADS-PL |  | Negative association of CPRS hyperactivity score with SF (p=0.02) & zinc levels (p=0.005) |  |
| Oner et al  2012 *a* [28] | Turkey | 713  ADHD | 613  100 | 7-15  9.1 | SF**  (N/A) | Hb  MCV  RDW | CPRS  CTRS  K-SADS-PL |  | Inverse correlation between CPRS hyperactivity score & SF. |  |
| Oner et al  2012 *b* [42] | Turkey | 345  ADHD | 302  43 | 6-15  9.1 | SF**  (<12 ug/L) | ---- | CPRS  CTRS  K-SADS-PL |  | Inverse correlation of baseline CPRS hyperactivity score & SF |  |
| Doom et al  2015 [11]  A case series embedded in a case control study assessing ADHD & IQ in IA children | Global | 69  IA children | ---- | ----  62.1 mo | SF  (<12 ug/L)  TIBC  TSAT | Hb  MCV | ECI-4  HBQ |  | More severe ADHD symptoms (and lower IQ) was found in IA children who had more severe ID at adoption. |  |
| **Cohort study**  **N=1** | | | | | | | | | | |
| Doom et al  2018 [26] | Chile | 1018  Adoles-cents | 519  499 | 11-17  14.3 | SF  (<12 ug/L) | FEP  Hb  MCV | CBCL  YSR | Positive association between self-reported ADHD & ID in infancy |  |  |
| **Cross-sectional study**  **N=1** | | | | | | | | | | |
| Ozturk et al  2020 [37] | Turkey | 205  Total  106  ADHD  99  ADHD-NOS | 137  68 | ----  ----  ----  9.93  ----  10.9 | SI**  SF**  (N/A) | Hb | T-DSM-IV-S | Significant negative correlation between SF & the total number of psychiatric diagnoses (p=0.001) | Significant negative correlation between SI and ADHD HA sub-scores (0.027), & SF & ADHD AD sub-scores (p=0.011) |  |

| **S3 Table**  **ADHD & ID no association**  **N=8** | | | | | | | | | |
| --- | --- | --- | --- | --- | --- | --- | --- | --- | --- |
| **Case control studies**  **N=4** | | | | | | | | | |
| **Ref.** | **Country** | **Study population** | | | **ID markers** | | **ADHD Instrument** | **Results** | |
|  |  | Cases  Controls  (n) | Male  Female  (n) | Age (y)  Range  Mean | Iron-  related  (cut-off values; N/A= no cutoff value specified) | RBC-related |  | ID & ADHD | ID & ADHD severity |
| Millichap et al  2006 [49] | USA | 68  ADHD  27 controls | 54  14  20  7 | 5-16  ----  4-14  9.5 | SF  (< 22 ug/L (**♂)**;  < 10 ug/L (♀)) | Hb  MCV | Unspecified parent & teacher questionnaires | No difference in mean SF between ADHD & controls | No difference between ADHD symptom severity in children with SF <20 ng/mL and >20 ng/mL |
| Menegassi et al  2010 [45] | Brazil | 41  ADHD  21 controls | 31  10  15  6 | 6-15  8.9  6-15  8.9 | SF  (12-15ng/mL; ≤ 30ug/L & ≤ 45ug/mL)  SI  TF | Hb  MCHC  MCV  RDW | K-SADS-E  SNAP-IV | No difference between iron & RBC markers |  |
| Abou-Khadra et al 2013 [46] | Egypt | 41  ADHD  62 controls | 35  6  43  19 | 6-12  8.03  6-12  8.60 | SF  (<30 ug/L) | ---- | CPRS |  | Children with SF <30 ng/mL showed no differences in ADHD subscale scores compared to those with SF >30 ng/mL |
| Donfrancesco et al 2013 [44] |  | 101 ADHD  93 controls | 92  9  82  11 | 6-14  107.3 mo  6-14  110.0 mo | SF  (<30 ug/L)  SI | Hb  MCV | K-SADS-PL  ADHD-RS | No difference between SF in ADHD & controls | No correlation between SF & ADHD severity and IQ |
| **Case series**  **N=3** | | | | | | | | | |
| Lahat et al  2011 [47] | Israel | 113  ADHD | 87  26 | 6-15  8.8 | SF  (<20 ug/L)  SI | Hb | CPRS  CTRS |  | Weak inverse correlation between CRS scores and SF |
| Magula et al  2019 [50] | South Africa | 245 | 196  49 | 6-18  ---- | SF  (<30 ug/L)  SI | ---- | ---- | No association between ADHD & ID |  |
| Unal et al  2019 [48] | Turkey | 100  ADHD | 72  28 | 6-12  9.01 | SF  (<12 ug/L) | Hb**  MCV  RDW | CPRS  K-SADS-PL |  | No correlation between SF or Hb & CPRS scores. Inverse correlation between Hb & learning problem/anxiety sub-scales |
| **Cohort study**  **N=1** | | | | | | | | | |
| Romanos et al  2013 [43] | Germany | 2805 | 1429  1376 | ----  10 | SF  (N/A) | ---- | SDQ |  | No inverse correlation between SF & hyperactivity/  Inattention SDQ sub-scale scores. |
