# Supplementary material for: Iron deficiency and common neurodevelopmental disorders—A scoping review
Source: PLoS One. 2022 Sep 29;17(9):e0273819. doi: 10.1371/journal.pone.0273819 (PMC9522276; doi:10.1371/journal.pone.0273819)
Supplement: S2 File — Studies investigating the association of ASD and ID. (DOCX) [file pone.0273819.s003.docx]

**S4, S5, S6 Tables. Studies investigating the association of ASD and ID**

** Indicates a statistically significant association; ---- indicates no information provided. Abbreviations: AuBC: Autism Behaviour Checklist; AbBC: Aberrant Behaviour Checklist; ADI-R: Autism Diagnostic Interview-Revised; ADOS: Autism Diagnostic Observation Schedule; CARS: Childhood Autism Rating Scale; EAR: estimated average requirement; GDD: global developmental delay; Hb- hemoglobin; Hct: hematocrit; ID: iron deficiency; IDA: iron deficiency anemia; MCV: mean corpuscular volume; MCHC: mean corpuscular hemoglobin concentration; RDW: red cell distribution width; SF: serum ferritin; SI: serum iron; TF: transferrin; TIBC: total iron binding capacity.

| **S4 Table**  **ASD & ID positive association**  **N=3** | | | | | | | | | | |
| --- | --- | --- | --- | --- | --- | --- | --- | --- | --- | --- |
| **Case control studies**  **N=2** | | | | | | | | | | |
| **Ref.** | **Country** | **Study population** | | | **ID markers** | | **ASD Instrument** | **Results** | |  |
|  |  | Cases  Controls  (n) | Male  Female  (n) | Age (y)  Range  Mean | Iron-  related  (cut-off values; N/A= no cutoff value specified) | RBC-related |  | ID & ASD | ID & ASD severity |  |
| Bener et al  2017 [59] | Qatar | 308  ASD  308 controls | 153  155  137  171 | 3-8  5.39  3-8  5.62 | SF**  (<12 ug/L (6mo-5yrs); 15 ug/L *or* > 30 ug/L if CRP ≥10 mg/L (>5 yrs))  SI** | Hb**  Hct**  MCH  MCV**  RDW | ADOS | SI lower in ASD (p=0.003). Hb, Hct, SF higher in controls (p<0.001) |  |  |
| Gunes et al  2017 [51] | Turkey | 100  ASD  100  Controls | 84  16  71  29 | 4-11  8.36  8-14  11.0 | SF  (<10 ug/L (preschool); <12 ug/L (school-aged)) SI** | Hb**  Hct**  MCV**  RDW | AbBC  AuBC  CARS | Lower Hct, Hb, SI, MCV in ASD | More severe ASD associated with lower Hb (p<0.05). Inverse correlation of Hct & CARS, AuBC, & AbBC scores (p<0.05) |  |
| **Case series**  **N=1** | | | | | | | | | | |
| Dosman et al  2006 [17] | Canada | 96  ASD | 78  18 | 1-10  5 | SF**  (< 10 ug/L (1-5 yrs);  < 12 ug/L (≥6yrs)) | Hb  MCV | ---- | Low SF in 1/12 1-2 year olds, 7/49 3-5 year olds, 7/35 6-10 year olds. 44/91 had low MCV & 17/91 had low Hb | Significant correlation between lower SF and higher ADOS communication scores (p=0.005) |  |

| **S5 Table**  **ASD & ID no association**  **N=3**  **Case control study**  **N=1** | | | | | | | | | |  |
| --- | --- | --- | --- | --- | --- | --- | --- | --- | --- | --- |
| **Ref.** | **Country** | **Study population** | | | **ID markers** | | **ASD Instrument** | **Results** | |  |
|  |  | Cases  Controls  (n) | Male  Female  (n) | Age (y)  Range  Mean | Iron-  related(cut-off values; N/A= no cutoff value specified) | RBC-related |  | ID & ASD  prevalence | ID & ASD  Severity |  |
| Liu et al  2016 [52] | China | 154  ASD  73  Controls | 141  13  67  6 | ----  5.2  ----  4.8 | SF  (N/A) | Hb | CARS | More frequent ID in the ASD group. ASD group had severe picky eating, resistance to new foods, & low micronutrient consumption compared to controls | No statistically significant correlation between SF & severity of ASD-related symptoms/CARS score | |
| **Case series**  **N=2** | | | | | | | | | |  |
| Dosman et al  2007 [18] | Canada | 33  ASD | 27  6 | 2-10  6 | SF  (< 10 ug/L (preschoolers);  < 12 ug/L (school aged))  sTfR | Hb  MCV | ADI-R  ADOS |  | No correlation between SF and baseline ADOS or ADI-R scores at baseline of treatment study investigating the effect of iron supplementation on sleep | |
| Bilgiç et al  2010 [53] | Turkey | 31  ASD | 27  4 | 1-5  40.26 mo | SF  (<10ug/L)  SI  TIBC | Hb  MCV | AbBC  AuBC  CARS | Low SF in 32.3% | No correlation between ID & symptom severity (p<0.05) | |

| **S6 Table**  **Studies describing the prevalence of ID in ASD**  **N=4** | | | | | | | | |
| --- | --- | --- | --- | --- | --- | --- | --- | --- |
| **Ref.** | **Country** | **Study population** | | | **ID markers** | | **ASD Instrument** | **Results** |
|  |  | Cases  (n) | Male  Female  (n) | Age (y)  Range  Mean | Iron-  related  (cut-off values; N/A= no cutoff value specified) | RBC-related |  | ID & ASD |
| Latif et al  2002 [54] | Wales | 96  Total  52  Autism  44  Asperger | ----  42  10  41  2 | ----  ----  1-8  ----  3-13  ---- | SF  (<12 ug/L) | Hb | ---- | Low SF in 12/23 with autism & 3/22 with Asperger. IDA in 6 with autism & 2 with Asperger |
| Hergüner et al  2012 [16] | Turkey | 116  ASD | 95  21 | 3-16  8.5 | SF  (< 10 ug/L (preschoolers); < 12 ug/L (school aged)) SI | Hb  Hct  MCV  RDW | ---- | Prevalence of ID & IDA 24.1% & 15.5% |
| Reynolds et al  2012 [56] | USA | 222  ASD | 193  29 | 2-11  5.3 | SF  (<12 ug/L)  SI  TIBC  TSAT | Hb | ADOS | Low SF (<12 ug/L) in 8%; low SF & TSAT in 1%; IDA in 1 child. Iron intake below EAR in <2% |
| Sidrak et al  2014 [55] | Australia | 122  ASD and/or GDD | 91  31 | 1-11  3 | SF  (<10ug/L)  Iron sat | Hb  MCV | ADOS  CARS  Griffiths Developmental Scales Assessment | Iron depletion in 2.5%; ID in 6.6%; IDA in 4.1%. Children with ASD with/without GDD had an iron depletion prevalence of 3.0%, ID of 7.5%, & IDA of 3.0%. Children with GDD without ASD had an iron depletion prevalence of 1.8%, ID & IDA was 5.5% |
