# Supplementary material for: Iron deficiency and common neurodevelopmental disorders—A scoping review
Source: PLoS One. 2022 Sep 29;17(9):e0273819. doi: 10.1371/journal.pone.0273819 (PMC9522276; doi:10.1371/journal.pone.0273819)
Supplement: S3 File — Treatment studies investigating the effect of iron supplementation in ADHD and ASD. (DOCX) [file pone.0273819.s004.docx]

**S7 and S8 Tables. Treatment studies investigating the effect of iron supplementation in ADHD and ASD.**

ADHD-RS: ADHD Rating Scale; CGI-S: Clinical Global Impression Severity; CPRS: Conner’s Parent Rating Scale; CSI-4: Child Symptoms Inventory-4; CTRS: Conner’s Teacher Rating Scale; Hb: hemoglobin; IBC: iron binding capacity; ID: iron deficiency; IV: intravenous; MCV: mean corpuscular volume; MPH: methylphenidate; RCT: randomized controlled trial; SF: serum ferritin; SI: serum iron; SNAP-IV: Swanson, Nolan, and Pelham-IV questionnaire; sTfR: soluble transferrin receptor; TF: transferrin; TSAT: transferrin saturation.

| **S7 Table**  **ADHD treatment studies**  **N=6** | | | | | | | | | |
| --- | --- | --- | --- | --- | --- | --- | --- | --- | --- |
| **Ref.** | **Country** | **Study population** | | | **ID markers** | | **ADHD Instrument** | **Method** | **Result** |
|  |  | Cases  Controls  (n) | Male  Female  (n) | Age (y)  Range  Mean | Iron-  related  (cut-off values; N/A= no cutoff value specified) | RBC-related |  |  |  |
| Tu et al  1994 [62] | Canada | 2 | 1  1 | 13-15  ---- | IBC  SF  (<20 ug/L)  SI  TSAT | Hb  MCV | ---- | Case report | Improvement of ID markers was associated with improvement of neuropsychiatric symptoms allowing discontinuation of psychotropic medications in 1 patient |
| Konofal et al  2005 [63] | France | 1 | 1  0 | 3  ---- | SF  (<15 ug/L) | Hb | CPRS  CTRS | Case report | After 8 months of treatment, SF increased from 13 ng/mL to 102 ng/mL. CPRS/CTRS scores were 30 & 32 at baseline, and decreased to 19 & 13, respectively. |
| Konofal et al  2008 [15] | France | 17  Treatment  5  Controls | 14  3  3  2 | 5-8  5.7  5-8  6.4 | SF  (<30 ug/L)  SI  sTfR  TF | Hb  MCV | ADHD RS  CPRS  CTRS | RCT  Jadad: 3/5 | After 12 weeks, a significant decrease in ADHD RS (p<0.008) & mean CGI-S (p<0.01) in treatment group, but not placebo. No significant improvement on CPRS/CTRS |
| Qubty et al  2014 [65] | USA | 3  Children with dev. delay and/or cognitive imp. | 2  1 | 1-12  ---- | SF  (N/A) | Hb  MCV | ---- | Case report | Iron supplementation resulted in improvement of SF and developmental markers in all of them. In the ADHD patient, IV iron sucrose (100 mg) led to an increase in SF from 8-23 ng/mL and of hyperactive symptoms and pica |
| Panahandeh et al  2017 [61] | Iran | 21  treatment  21  controls | 16  5  19  2 | 5-15  8.95  5-15  7.57 | SF  (<30 ug/L) | Hb | CSI-4 | RCT  Jadad: 2/5 | Both groups had ADHD and were treated with MPH. The treatment group received additional iron supplementation. CSI-4 scores decreased significantly at month 2 in both groups (p<0.001). CSI-4 scores were significantly lower in the treatment group (p<0.05). No significant differences in the mean total CSI-4 scores and inattentive & hyperactive/impulsive subtypes between sex groups at baseline & after 2 months of treatment (p<0.05) |
| Ghogare et al  2020 [64] | India | 6  ADHD | 5  1 | 8-15  ---- | SI  SF  (<22ug/L)  TIBC | Hb  MCH  MCHC  MCV  RDW | SNAP-IV | Case series | SNAP IV parent & teacher scores decreased after 3 months of treatment with oral iron supplements |

| **S8 Table**  **ASD treatment study**  **N=1** | | | | | | | | | |
| --- | --- | --- | --- | --- | --- | --- | --- | --- | --- |
| **Ref.** | **Country** | **Study population** | | | **ID markers** | | **ASD Instrument** | **Method** | **Result** |
|  |  | Cases  Controls  (n) | Male  Female  (n) | Age (y)  Range  Mean | Iron-  related  (cut-off values; N/A= no cutoff value specified) | RBC-related |  |  |  |
| Reynolds et al  2020 [66] | Canada/  USA | 9  11 | 6  3  11  0 | 2-10 6.0  2-10  5.7 | SF  (>17 & <50 ug/L) | Hb  Hct  MCV | ADOS  SNAP-IV | RCT  Jadad: 5/5 | No significant differences in SNAP-IV scores between the two groups. |
